# Self-Assembling Peptide Surfactants A_6_K and A_6_D Adopt α-Helical Structures Useful for Membrane Protein Stabilization

**DOI:** 10.3390/membranes1040314

**Published:** 2011-10-21

**Authors:** Furen Zhuang, Kamila Oglęcka, Charlotte A. E. Hauser

**Affiliations:** Institute of Bioengineering and Nanotechnology, 31 Biopolis Way, The Nanos #04–01, Singapore 138669; E-Mails: frzhuang.yrp@ibn.a-star.edu.sg (F.Z.); koglecka@ibn.a-star.edu.sg (K.O.)

**Keywords:** self-assembling peptides, membrane proteins, peptide surfactants, peptide structure, GPCRs

## Abstract

Elucidation of membrane protein structures have been greatly hampered by difficulties in producing adequately large quantities of the functional protein and stabilizing them. A_6_D and A_6_K are promising solutions to the problem and have recently been used for the rapid production of membrane-bound G protein-coupled receptors (GPCRs). We propose that despite their short lengths, these peptides can adopt α-helical structures through interactions with micelles formed by the peptides themselves. These α-helices are then able to stabilize α-helical motifs which many membrane proteins contain. We also show that A_6_D and A_6_K can form β-sheets and appear as weak hydrogels at sufficiently high concentrations. Furthermore, A_6_D and A_6_K together in sodium dodecyl sulfate (SDS) can form expected β-sheet structures via a surprising α-helical intermediate.

## 1. Introduction

Self-assembling peptides are a class of biomolecules that assemble spontaneously on their own into ordered nano- or even supramolecular structures. They are useful in many aspects of biomedicine and bionanotechnology, with potential applications in tissue engineering and drug delivery, amongst many others [1]. These self-assembling peptides can be linked with lipids, sugars and other compounds or functional groups to allow greater flexibility for different uses, making them a preferred material to the relatively less reactive carbon nanotubes [2,3,4].

A subset of such peptides are the short self-assembling surfactant peptides which consist of a hydrophilic head with a charged polar amino acid (e.g., aspartic acid or lysine) and a hydrophobic tail of two or more hydrophobic amino acids. These peptides are similar to natural phospholipids and self-assemble into nanofibers or nanovesicles [5,6,7,8,9]. The short length of these peptides (ranging from as low as three amino acids), allows for cheaper production of molecular scaffolds, which usually have prohibitively high costs. Here we discuss the self-assembling properties of two such peptides, Ac-AAAAAAD-COOH (A_6_D) and Ac-AAAAAAK-CONH_2_ (A_6_K), which incidentally allow them to be used in the cell-free production of functional membrane proteins, for elucidation of their structures [10].

Elucidating the structures of membrane proteins have proved to be very challenging: Out of over 72,000 structures in the Protein Data Bank, only 280 are unique membrane proteins. This is mainly due to the difficulties involved in: (1) producing adequately large quantities of the functional protein, and (2) stabilizing them for a sufficiently long period of time. Over the years, peptide surfactants such as A_6_D and A_6_K have emerged as promising candidates to overcome these bottlenecks in studying membrane protein and function [11,12,13,14].

A_6_D and A_6_K can both be used to extract integral proteins. A_6_D and A_6_K have individually been shown to be capable of extracting the membrane-integrated flavoenzyme glycerol-3-phosphate dehydrogenase (GlpD) from membranes of *E. coli*, to similar extents (≥50%) as conventional non-ionic surfactants [15], and with comparable retained specific activity (∼15–20%).

Additionally, A_6_D and A_6_K possess the ability to interact with and strongly stabilize membrane proteins. A_6_K, for example, can stabilize Photosystem I for a period of more than two months in aqueous media [14], while A_6_D has been shown to significantly enhance the thermal stability of the GPCR rhodopsin in the absence of lipids [13].

Recently, A_6_D and A_6_K have also been used in commercial *E. coli* cell-free systems to produce milligram quantities of soluble G protein-coupled receptors (GPCRs), such as the human formyl peptide receptor and the human trace amine-associated receptor, for analysis with X-ray crystallography [10].

Key to the ability of A_6_D and A_6_K to stabilize these membrane proteins is their ability to spontaneously form α-helices which can stabilize the α-helical motifs of many membrane proteins. The aim of this paper was to study how these peptides can self-assemble into α-helices and stabilize membrane proteins.

However, analyzing the assembly process of A_6_D and A_6_K is challenging due to the fact that imaging techniques such as Atomic Force Microscopy (AFM) and cryo Transmission Electron Microscopy (TEM) can only image the larger tertiary structures and are not suitable for the observation of finer secondary structural changes. While Circular Dichroism (CD) analyses offer a method to monitor and quantify the secondary structural changes in real time, it is precluded by the increasing light attenuance of the sample at higher concentrations and as it assembles. To overcome this limitation, we conducted CD experiments with very low peptide concentrations below 10 mM.

## 2. Materials and Methods

### 2.1. Chemicals

Peptides A_6_D and A_6_K of ≥98% purity were purchased from GL Biochem (Shanghai, China) and used without further purification. The concentration of peptide stock solutions in MilliQ water was estimated by weight due to the lack of aromatic residues that could be utilized for spectroscopic concentration determination. The pH of the peptide solutions was not adjusted. Sodium dodecyl sulfate (SDS) was purchased from Ameresco Solon Inc., Ohio, USA.

### 2.2. Circular Dichroism (CD) Measurements

Circular dichroism measurements were performed on an Aviv 410 spectropolarimeter using a rectangular 1 mM quartz cell with a fitted cap. Temperature melts were conducted by gradually increasing the temperature in a step-wise fashion and then inverting the process. The investigated temperatures ranged from 4–90 °C (in the following steps: 4 °C, 6 °C, 10 °C, 15 °C, 20 °C, 25 °C, 37 °C, 40 °C, 50 °C, 70 °C, 90 °C and then run again at 4 °C and 25 °C) or 20–90 °C (in steps of: 20 °C, 25 °C, 37 °C, 40 °C, 50 °C, 70 °C, 90 °C and then cooled while acquiring data at the same temperatures). Prior to data acquisition, samples were equilibrated for 1 min at each temperature. Data acquisition was performed in steps of 0.5 nm between 260–190 nm, except when kinetics was fast and a pitch of 1 nm between 270–185 nm was implemented instead. The averaging time for each data point was either 20 or 4 s—depending once more on the observed kinetics. The use of a long averaging time was only justified when no time-dependent effects could be observed during the full experimental time (ranging between 8 to 25 h). Between two and 100 scans were performed at each environmental state depending on the effects being studied. All spectra were baseline corrected with blanks corresponding to appropriate temperatures, but averaging was only performed when secondary structure did not change over time. The concentration of peptides in MilliQ water ranged between 0.25–10 mM. Ultimately, all spectra were converted into mean residue ellipticity (MREs) using Equation 1:

(1) $${\left[\theta \right]}_{\lambda }=\frac{\theta_{\mathrm{Obs}}}{\left(10\times l\times c\times (n-1)\right)}$$

where [*θ*]λ is the MRE at wavelength λ in deg cm^2^ dmol^−1^, *l* is the path length in cm, *c* is the concentration in M and (*n* − 1) designates the number of peptide bonds in the studied peptide.

Increases in the amount of helical content can then be judged from a deepening of the CD signal at 222 nm, while an increase in β-sheet content can be judged from a deepening of the CD signal at 217 nm. Quantification of the amount of α-helical and β-sheet contents was however not done, due to the absence of aromatic residues, which prevented the precise determination of peptide concentration needed for quantification.

### 2.3. Field Emission Scanning Electron Microscopy (FESEM) Studies

Samples were frozen at −80 °C or preferably shock frozen in liquid nitrogen and vacuum dried. Subsequently they were fixed onto a sample holder using conductive tape and sputtered with platinum from both the top and the sides in a JEOL JFC-1600 High Resolution Sputter Coater. The coating current used was 30 mA, and the process lasted for 60 s. The surface of interest was then examined with a JEOL JSM-7400F field-emission scanning electron microscopy system using an accelerating voltage of 5–10 kV.

## 3. Results and Discussion

### 3.1. Secondary Structures of A_6_D and A_6_K Peptide Surfactants in SDS

The peptides were first tested in SDS to ascertain if these ultrashort peptides could form α-helices. Like trifluoroethanol (TFE), SDS in concentrations above its CMC (∼8.2 mM) is widely known to induce α-helical conformations in numerous polypeptides [16,17,18,19,20,21,22].

**Figure 1:**
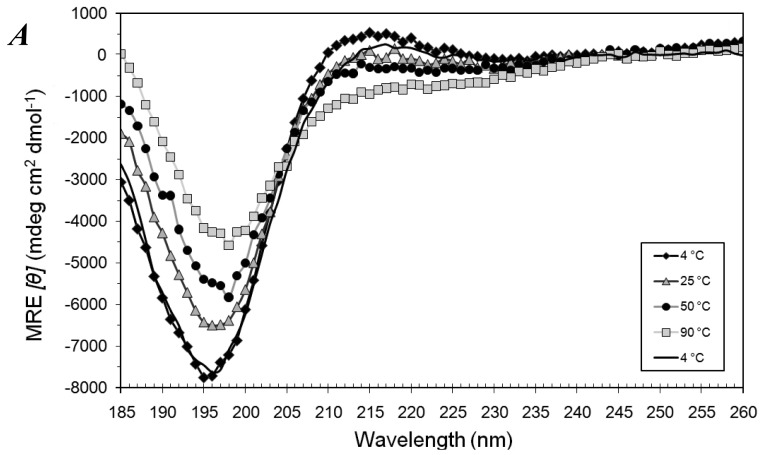

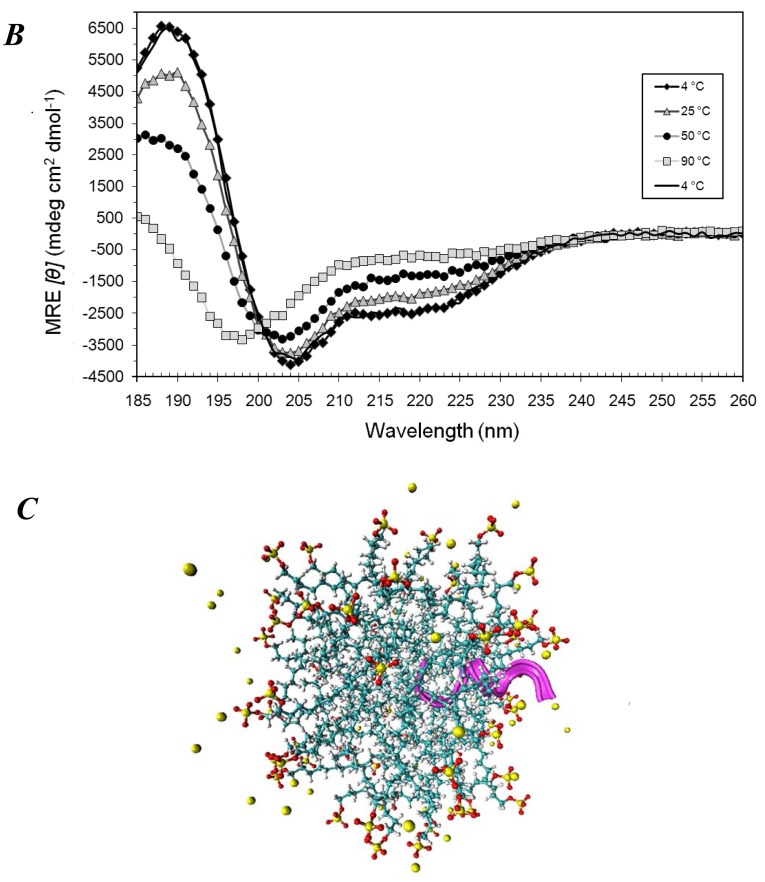
(**A**) Mean residue ellipticity (MRE) of 0.4 mM A_6_D in 13 mM sodium dodecyl sulfate (SDS) when heated stepwise from 4 °C to 90 °C and then cooled back to 4 °C. (**B**) MRE of 0.4 mM A_6_K in 13 mM SDS when heated stepwise from 4 °C to 90 °C and then cooled back to 4 °C. Strong negative MRE at 208 nm and 222 nm indicated a predominantly α-helical structure, which could be refolded after cooling down from 90 °C. (**C**) A depiction of SDS micelle interacting with A_6_K peptide surfactant in water. The A_6_K peptide is labeled in pink with an α-helical structure. The hydrophobic tails of SDS molecules are in green and the hydrophilic head of SDS is in yellow (sulfur) and red (oxygen).

Samples of A_6_D and A_6_K (0.4 mM) were heated from 4 °C to 90 °C and then cooled back to 4 °C, in the presence of 13 mM SDS. While A_6_D remained predominantly random coiled (Figure 1A), A_6_K astonishingly formed well-defined α-helices as judged from the CD spectra (Figure 1B).

Typical α-helices have 3.6 residues per turn, and are usually formed by longer peptides. Being 7 residues long, A_6_K can hardly form a full turn of the α-helix and can only form 4 intra-molecular hydrogen bonds. Its surprising ability to form a stable α-helix makes it one of the shortest α-helices observed.

The length of an A_6_K molecule is about 1.1 nm, comparable to the length of an outstretched SDS molecule, which is 1.67 nm [23]. Given the structural similarity of A_6_K to amphiphilic SDS molecules, it is very likely that A_6_K interacted with SDS molecules to form micellar structures (Figure 1C). This conformational change occurred instantly when SDS was added to A_6_K and the partial unfolding of the helix due to heating from 4 °C to 90 °C exhibited complete thermal reversibility when cooled to 4 °C.

A_6_D, however, did not show any observable secondary structural transitions when 13 mM SDS was added to it at 25 °C. This is probably because the positively-charged lysine headgroup of A_6_K interacts better with the anionic SDS molecules, as compared to the negatively-charged Asp headgroup of A_6_D.

A major concern in the interpretation of the results was that some measurements were made below the Krafft point of SDS, 22 °C. Above this Krafft point, the solubility of SDS greatly increases in aqueous systems and is regarded as the melting temperature of the hydrated SDS solid. Hence below the Krafft point, SDS does not form micelles but hydrated crystals. In the experiment above, SDS was added to the peptides at 25 °C and then rapidly cooled to 4 °C. Micelles were formed and probably stabilized by the presence of the peptides, and hence remained intact even at 4 °C. Low temperatures were used in this case in an attempt to slow down the kinetics of the peptides and SDS interaction, to a suitably low rate for observation. The trend of the CD signal below and above the Krafft point agree with each other, indicating that the Krafft point probably did not affect the peptide-SDS interactions very significantly.

However, if SDS at 4 °C was added to the peptide, no structural induction was seen. This supports the hypothesis that SDS micelles were needed for the structural induction of A_6_K to α-helices.

### 3.2. Secondary Structures of A_6_D and A_6_K Peptide Surfactants in Water

**Figure 2:**
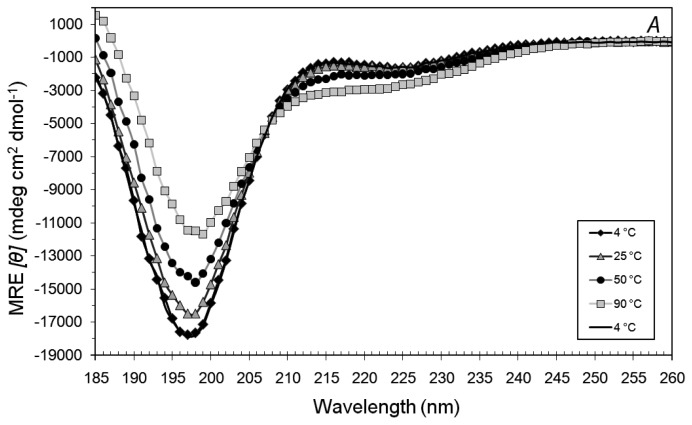

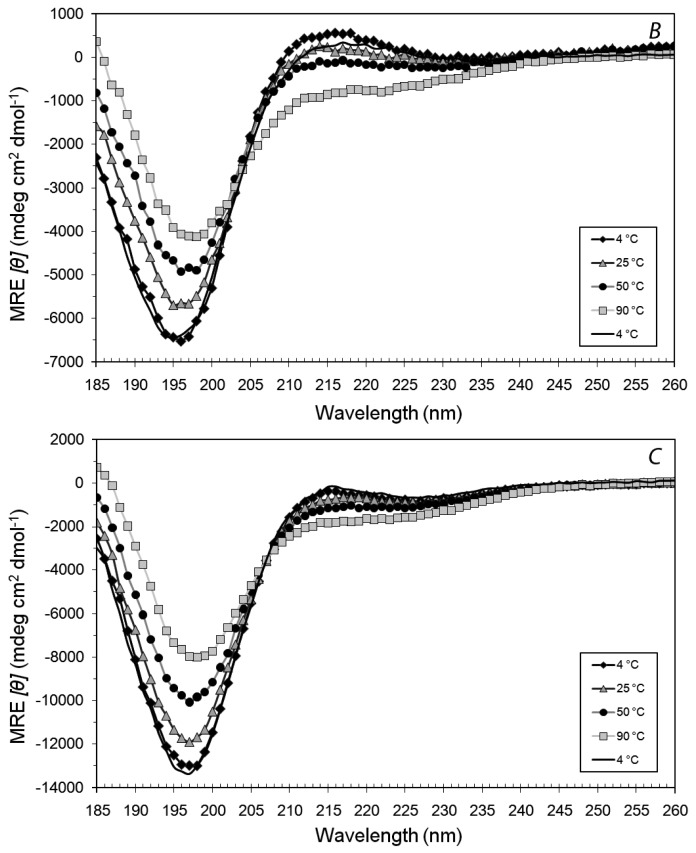
Mean residue ellipticity (MRE) of (**A**) 0.4 mM A_6_K, (**B**) 0.4 mM A_6_D and (**C**) 0.2 mM A_6_K and 0.2 mM A_6_D, in water when heated stepwise from 4 °C to 90 °C and then cooled back to 4 °C. Peptides appear to remain random-coiled and the spectra in (C) appears to be merely a combination of 2A and 2B, suggesting that structural changes due to the interaction of A_6_K and A_6_D was not very significant.

Heating samples of each peptide (0.4 mM) from 4 °C to 90 °C and then cooling back to 4 °C in water revealed a mostly random coiled conformation with a small proportion of helical motifs which increased at higher temperatures. The secondary structural changes were fully reversible (Figure 2A and Figure 2B), suggesting weak interactions.

**Figure 3:**
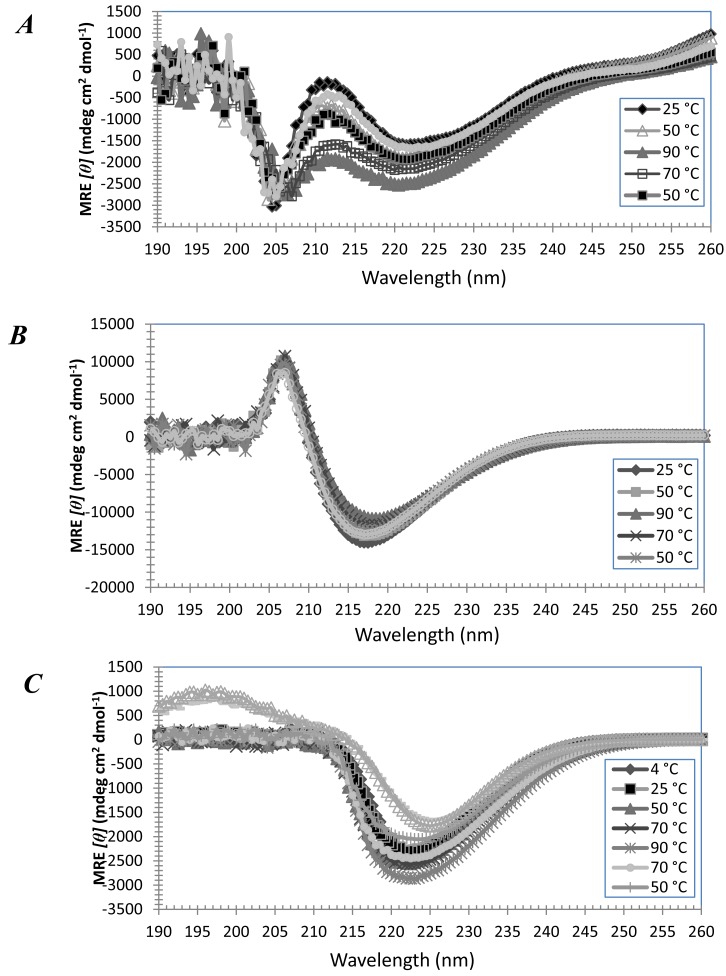

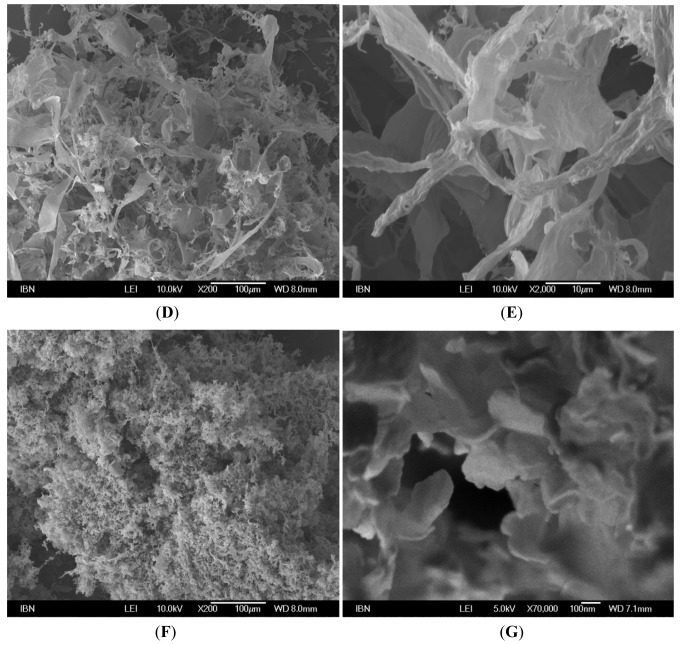
(**A**) Mean residue ellipticity (MRE) of 2.5 mM A_6_K in water. The strong negative MRE at 208 nm and 222 nm indicates a predominantly α-helical structure. (**B**) MRE of 2.5 mM A_6_D, and (**C**) MRE of 10 mM A_6_K in water. Strong negative MRE around 217 nm indicates β-sheet structures. (**D**,**E**) FESEM images of 2.5 mM A_6_K and (**F**,**G**) 2.5 mM A_6_D, without SDS.

At higher concentrations of 2.5 mM, A_6_K formed α-helices which were not fully reversible (Figure 3A), but A_6_D remained predominantly random-coiled (data not shown), reflecting similar results to the previous experiments in 13 mM SDS. This is probably possible because the amphiphilic peptides at higher concentrations can form micelles in place of SDS, to induce α-helical structures on the peptides.

At even higher concentrations, both A_6_K and A_6_D can form β-sheets (Figure 3B and 3C). Under FESEM, these are seen as spongy, cavity-containing membranous structures which allow large quantities of water to be held (Figure 3D–G). These peptide solutions hence tend to appear as hydrogels. The process of assembly from α-helices to β-sheets is however not yet clear.

### 3.3. Secondary Structures of A_6_D and A_6_K in Combination, in Water

We were then interested in how the two peptides would interact when placed together, given their complementary structures. When 0.2 mM A_6_D and 0.2 mM A_6_K were combined and subjected to the same temperature treatment, the resultant CD spectra (Figure 2C) appears to be a combination of the spectra observed in Figure 1A and Figure 1B. This suggests that the combined A_6_D and A_6_K structures are similar to their individual ones.

### 3.4. Secondary Structural Changes of a Combination of A_6_D and A_6_K in SDS

**Figure 4:**
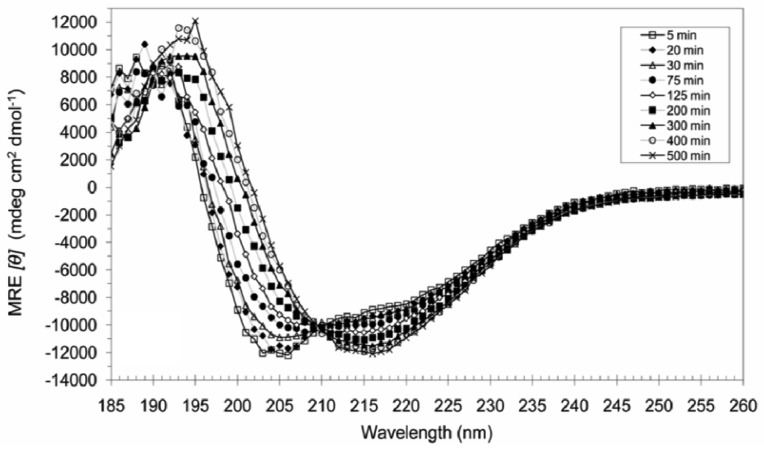
Scans of 0.2 mM A_6_D and 0.2 mM A_6_K in 20 mM SDS monitoring the structure from 5 min to about 8.5 h at 50 °C. The combination initially showed primarily α-helical character, but adopted β-sheet features with time. The initial conversion rates were fast, but slowed down as the structural conversion progressed.

However, when 20 mM SDS was added to a mixture of 0.2 mM A_6_D and 0.2 mM A_6_K at 50 °C and incubated for 8.5 h, anti-parallel β-sheets (Figure 4A) were formed which were very stable and could not be reversed by heating to 90 °C, cooling to 4 °C, sonication (20 min) or subsequent SDS additions.

Interestingly, the peptides did not proceed directly from random coil to β-sheets, but were instead formed through an unstable α-helical intermediate which progressed to the more stable β-sheet conformation with kinetic rates which increased with temperature. At temperatures of 25 °C and below, the kinetic rates of α → β conversion occurred over days, while at 37 °C and above, the conversion could be clearly observed after a timescale of hours.

A recent study has reported on even smaller tri- to hexapeptides that could adopt helical conformations under forced aprotic conditions [24,25]. More recently, we were able to observe α-helical structures with ultrashort linear tri- to hexapeptides aliphatic peptides even under aqueous conditions [26]. These peptides used the α-helical confirmation as an intermediate transition structure toward their arrangement to a final β-turn structure. It is likely that A_6_D and A_6_K similarly requires aprotic conditions to transit to α-helical and β-sheet conformations. As mentioned earlier, at higher concentrations, A_6_K alone without SDS addition can also form α-helices and β-sheets, probably also due to aprotic environments created by surfactant A_6_K molecules.

Furthermore, since structural induction of 0.2 mM A_6_D and 0.2 mM A_6_K to β-sheets does not occur in lower concentrations of SDS (e.g., 5 mM), it is likely that intact micelles are required to induce these conformational changes.

When 0.2 mM A_6_D and 0.2 mM A_6_K was mixed in combination with 20 mM SDS at low temperatures, A_6_K was probably incorporated into SDS micelles with far greater efficiency than A_6_D, leading to structural induction in A_6_K but not A_6_D. It has been shown that an α-helix motif cannot be followed immediately by a β-strand motif in a covalently-linked chain due to steric clashes [27], and since these heptapeptides are too short to incorporate the necessary linkers to maintain two partial conformations, it is possible that instead of having peptides of partial α- and β-conformations, the two peptides are present in two different conformations and the CD spectra are simply average representations.

As temperature increases, the SDS micelles become destabilized, allowing A_6_D to interact with the less SDS-shielded A_6_K, probably initiated by electrostatic attraction between the oppositely-charged headgroups. The charge neutralization following the formation of an ionic bond allows the hydrophobic driven incorporation of peptide dimers into micelles, with a subsequent conversion into β-sheets. The presence of SDS provides the aprotic environment required to overcome the activation energy needed to induce assembly.

## 4. Conclusions

Our study of the above two peptides hypothesizes a probable mechanism in which short surfactant peptides such as A_6_D and A_6_K could aid in the cell-free production of membrane proteins. The amphiphilic property of these peptides allows the formation of micelles that induce peptides to adopt α-helical conformations and stabilize these membrane proteins.

Furthermore, we describe a mechanism by which A_6_D and A_6_K could assemble into β-sheets via an α-helical intermediate, requiring an aprotic condition provided by the presence of SDS. We believe these assembly processes are not only relevant to such simple systems, but may also be involved in the amyloid fibril formation of many diseases such as Alzheimer's and Parkinson's. By studying these relatively simple systems, the assembly processes of these diseases may also be unraveled.
