# Correction: Self-Assembling Peptide Surfactants A_6_K and A_6_D Adopt α-Helical Structures Useful for Membrane Protein Stabilization. *Membranes* 2011, *1*, 314-326

**DOI:** 10.3390/membranes2020214

**Published:** 2012-05-02

**Authors:** Kamila Oglęcka, Furen Zhuang, Charlotte A. E. Hauser

**Affiliations:** Institute of Bioengineering and Nanotechnology, 31 Biopolis Way, The Nanos #04–01, Singapore 138669, Singapore; Email: kamila.oglecka@ntu.edu.sg (K.O.); fz230@cam.ac.uk (F.Z.)

We would like to request a correction to the author listing. The following changes should be made in respect to the original publication of this article [1].

Kamila Oglęcka ^†‡^ Furen Zhuang ^†^ and Charlotte A. E. Hauser *

Institute of Bioengineering and Nanotechnology, 31 Biopolis Way, The Nanos #04–01, Singapore 138669; Email: kamila.oglecka@ntu.edu.sg (K.O.); fz230@cam.ac.uk (F.Z.)

^†^ These authors contributed equally to this work.

^‡^ Present address: Nanyang Technological University, SBS-02s-85, 60 Nanyang Drive, Singapore 637551.

* Author to whom correspondence should be addressed; Email: chauser@ibn.a-star.edu.sg; Tel.: +65-6824-7108; Fax: +65-6478-9080.
